# Effects of Remifemin Treatment on Bone Integrity and Remodeling in Rats with Ovariectomy-Induced Osteoporosis

**DOI:** 10.1371/journal.pone.0082815

**Published:** 2013-12-09

**Authors:** Guangxia Cui, Huijie Leng, Ke Wang, Jianwei Wang, Sainan Zhu, Jing Jia, Xing Chen, Weiguang Zhang, Lihua Qin, Wenpei Bai

**Affiliations:** 1 Department of Obstetrics and Gynecology, Peking University First Hospital, Beijing, China; 2 Department of Orthopaedics, Peking University Third Hospital, Beijing, China; 3 Department of Anatomy and Embryology, Peking University Health Science Center, Beijing, China; 4 Statistics Office, Peking University First Hospital, Beijing, China; 5 Department of Stomatology, General Hospital of Armed Police, Beijing, China; University of Michigan Medical School, United States of America

## Abstract

This study aims to evaluate the effects of Remifemin (isopropanolic extract of *Cimicifuga Racemosa*) on postmenopausal osteoporosis. 120 female Sprague-Dawley rats were randomly assigned to four groups: sham surgery with vehicle, ovariectomy with vehicle, ovariectomy with estradiol valerate, or ovariectomy with Remifemin. Daily oral administrations of the vehicle, estradiol valerate, or Remifemin began 2 weeks after surgery and lasted to 4, 8, or 12 weeks. Ten rats in each group were sacrificed at each timestep with assessment of bone mineral density, trabecular bone structure, and biomechanical parameters of the femur and lumbar vertebra. Bone turnover markers were evaluated 12 weeks after surgery. Both drugs prevented bone density loss in the distal end of the femur and preserved the trabecular bone structure in both the lumbar vertebra and distal end of the femur following ovariectomy. Both drugs protected bone stiffness at the tested regions and reduced bone reabsorption in ovariectomized rats. The preventive effects of Remifemin against bone-loss can rival those of estradiol valerate if treatment duration is adequately extended. In conclusion, Remifemin may demonstrate equivalent effects to estradiol valerate in terms of preventing postmenopausal osteoporosis.

## Introduction

Osteoporosis, which places a considerable economic burden on both families and societies, is a global medical issue with an increasing woldwide incidence [1,2]. Osteoporosis is characterized by reduction and deterioration of the microarchitecture of bone tissue, consequently increasing bone frailty and susceptibility to fracture [3]. There are three types of osteoporosis: postmenopausal osteoporosis (PMO), age-related osteoporosis, and secondary osteoporosis [4]. Women are more prone to bone-loss than men following postmenopausal estrogen deficiency; thus, PMO is the most common type of osteoporosis [1]. 

The exact mechanism of PMO is not completely understood. It is commonly believed that estrogen deficiency promotes bone absorption over formation. Imbalance between bone formation and bone resorption eventually leads to bone loss and reduces the mechanical functions of bone [1,5-7]. As a result, treatment for PMO includes both the promotion of bone formation and the inhibition of bone reabsorption. Hormone replacement therapy (HRT) is typically used to promote bone formation. It is already established that HRT prevents fractures in postmenopausal women [8,9]; however, the benefits of HRT, in terms of preventing disease, long-term disability, and death in postmenopausal women, may be outweighed by the higher incidence of treatment-related thromboembolic complications [10]. In additon, HRT also increases the risk of breast and gynecological tumors [11,12], which significantly limits its clinical applications. Diphosphonate inhibits bone reabosorption. However, diphosphonate demonstrates mutiple adverse effects, such as mandible osteonecrosis, which is a major concern in clinical settings [13-15]. Selective estrogen receptor modulators (SERMs), which have evolved through multiple generations for the prevention and/or treatment of postmenopausal osteoporosis, have been associated with increased venous thromboembolic events and hot flushes[16]. Finding a safe and effective drug for the treatment of PMO is clinically important and has become an important area of research. 

Remifemin is a herbal medicine that is applied widely for alleviating menopausal symptoms, particularly vasomotor symptoms (hot flushes, sweatings and hot flush associated sleep disturbances) and, to some extent, menopausal anxiety, menopausal depressive moods, nervousness, etc [17-21]. It is worth noting that the commercially available isopropanolic formulation of Cimicifuga (Remifemin) has been widely studied and does not induce cytotoxic, mutagenic, carcinogenic, or teratogenic effects at doses much larger than the human therapeutic dose, and its safety and effectiveness have both been confirmed [22-26]. In addition to the effectiveness of treating menopausal symptoms, Nißlein et al reported that Remifemin can significantly diminish the urinary content of PYR and DPY (specific markers for bone loss) and the morphometric correlates of bone loss associated with ovariectomy in rats, demonstrating a certain bone-sparing effects [27]. We therefore evaluated the effects of Remifemin treatment more comprehensively on bone integrity and remodeling in rats with developing osteoporosis due to ovariectomy.

## Materials and Methods

### 1: Ethics Statement

All experimental procedures and protocols were approved by the Biomedical Ethics Committee of Peking University (the approval number: LA2012-82 ). All operation was performed under 1% sodium pentobarbital intraperitoneal injection anesthesia, and all efforts were made to minimize suffering.

### 2: Medications

The drugs used in this study included *Cimicifuga racemosa* (Remifemin^®^ tablets) and estradiol valerate (Bujiale^®^; 1 mg/tablet), both of which are commercially available. The manufacturers of these two drugs are Schaper & Brümmer GmbH & Co. KG (batch number 063471; Germany) and the Guandong branch of Bayer Healthcare Co, Ltd (batch number 026A 11), respectively. The active ingredients in each Remifemin tablet (0.018–0.026 mL liquid extract) correspond to, on average, 2.5 mg dry extract or 20 mg crude drug (extraction agent: 40% [v/v] isopropanol) [28].

The two drugs were prepared as follow: Remifemin and estradiol valerate tablets were suspended in saline and sonicated, and a homogeneous suspension was maintained by stirring [4]. The concentrations of Remifemin and estradiol valerate were 12 mg/mL (concentration of corresponding crude drug) and 0.2mg/mL, respectively.

### 3: Animal groups, treatment, and processing

One hundred and twenty 3-month-old female Sprague Dawley rats (weight: 250±10 g) were purchased from the Department of Laboratory Animal Science, Peking University Health Science Center, and raised there. Before surgery, all rats were housed in the laboratory and given one week to adapt to their surroundings, which were maintained at a constant temperature of 25°C and relative humidity of 50%. The room was maintained under a 12/12-hour artificial light/dark cycle. All rats were allowed free access to water and soy-free pelleted diet, which was used to eliminate the effects of phytoestrogen throughout the experiment [29]. 

Rats were randomly assigned to four groups: sham surgery with vehicle (SHAM; n = 30), ovariectomy with vehicle (OVX+NS; n = 30), ovariectomy with estradiol valerate (OVX+E; n = 30), and ovariectomy with Remifemin (OVX+iCR; n = 30). SHAM rats underwent bilateral laparotomy but the ovaries were left in place, while rats in the other three groups underwent bilateral ovariectomy via the ventral approach [28]. Both the sham surgeries and ovariectomies were performed under 1% sodium pentobarbital (40 mg/kg intraperitoneal injection) and sterile conditions. In accordance with a previously published study [27], vaginal smears were collected 3–10 days after surgery in order to observe the effects of the surgery. All groups (except the SHAM group) demonstrated successful ovariectomy. 

After 2 weeks of recuperation, all animals received the following treatments: SHAM, once-daily administration of 10 mL/kg physical saline; OVX+NS, once-daily administration of 10 mL/kg physical saline; OVX+E, once-daily administration of 0.8 mg/kg estradiol valerate; OVX+iCR, once-daily administration of 60 mg/kg crude drug. Drugs were administered everyday by lavage between 8:30 and 9:30 am. All rats were weighed prior to administration in order to adjust the drug dose.

At 4-, 8-, and 12-week postoperation, 10 rats were randomly selected from the SHAM, OVX+NS, OVX+E, and OVX+ iCR groups and sacrificed via an overdose of anesthesia. The right and the left femurs and lumbar vertebrate 2–4 (L2-L4) were dissected from the adjacent soft tissues, removed, and stored at -20°C in physiological saline for the subsequent assessment of bone mineral density (BMD), bone biomechanical quality, and trabecular bone structure. The second lumbar vertebrate was identified by counting down from the last thoracic vertebrate. Blood samples were taken from the angular vein at 12-week postoperation. Serum was prepared by centrifuging the collected blood samples (4000 rpm for 10 minutes at 4°C). All samples were stored at -80°C until further chemical analysis.

### 4: Measurment of bone mineral density

The BMD of ten rats at each group at 4-, 8- or 12-week postoperation was measured using a dual-energy X-ray absorptiometry (DXA) system (Discovery A; Hologic Inc., Boston, MA, USA) equipped with a high-resolution collimator. Areas 1 cm from the most proximal end of the femur (femoral proximal end) and 1 cm from the most distal end of the femur(femoral distal end), in addition to L2-L4, were used to assess BMD. BMD was calculated from the bone mineral content (BMC) values of the measured areas.

### 5: Trabecular bone structure testing

#### 5.1: Micro CT

Following the determination of bone mass, 6 rats were randomly selected from each group at 4-, 8- or 12-week postoperation. L3 and the right femoral distal end were used to evaluate the trabecular bone structure using Pre-Clinical Specimen microcomputed tomography (Micro CT, Inveon; Siemens Medical Solutions USA, Inc., Knoxville, TN, USA). The lumbar vertebrae were scanned to measure the entire length, while the femoral distal ends were scanned at 10 mm intervals, at a voxel size of 14 μm, 80 kV, 500 µA, and an exposure time of 1000 ms. The trabecular region was isolated from the cortical region in each 2D image by a manual contouring analysis. Model-independent 3D measurement methods were used to reconstruct and define the trabecular bone volume of interest (VOI) for the cancellous bone The following parameters were obtained by analyzing VOI: relative bone volume (BV/TV), trabecular number (TbN), trabecular spacing (TbSp), and trabecular thickness (TbTh).

#### 5.2 H&E staining

The left femoral distal ends of the above selected rats from each group at 12-week postoperation were used to study the trabecular histomorphology using H&E staining. The bone samples were removed and fixed in 4% neutral-buffered formalin for 24 hours succeed by a 3-week decalcification at 4 °C using a 10% ethylene diamine tetraacetic acid (EDTA) solution (PH 7.4). After decalcification, bone samples were dehydrated in ascending series of increasing concentrations of alcohol, infiltrated and embedded in paraffin. Bone samples were then cut to 7 μm coronal sections using a AO-820 rotary microtome (Southbridge, MA, USA) followed by a hematoxylin and eosin staining. Images were obtained using a Olympus BX51 microscope (Olympus, Japan).

### 6: Evaluation of the biomechanical properties of bone

Following the morphometric measurement of the bone tissue, the L3 and the right femoral distal ends of the 6 rats per group were selected and used to evaluate the biomechanical properties of these bone samples using the MTS Landmark System (Eden Prairie, MN,USA) separately. A low-speed diamond wheel saw was used to remove the spinous and transverse processes from L3, and the ends of the centrums were pruned to create two parallel surfaces for compression testing. The femoral distal ends were also processed to make sure the adjacent soft tissues were removed. After specimen preparation, each centrum (5 mm in height) and femoral distal end was fixed to the center of a steel plate, and a second plate was positioned along the longitudinal axis (L3) or perpendicular to the longitudinal axis (femoral distal end) with a constant displacement rate of 3 mm/minute or 2 mm/minute, respectively [30,31]. The specimens were loaded in compression until failure, and a load-displacement curve was calculated to assess stiffness (i.e., extent to which the specimen deformed under a particular load) and bone strength (i.e., ultimate load that factured the specimen). 

### 7: Detection of bone turnover markers in serum

The concentration of serum osteocalcin (OC) was measured using the rat ELISA kit (Biomedical Technologies Inc., Stoughton, USA). Procollagen type I N-terminal propeptide (PINP) and tartrate-resistant acid phosphatase 5b (TRACP 5b) levels were also determined using rat ELISA kits (IDS Ltd., Boldon, UK). 

### 8: Statistical analysis

Analyses were carried out using SPSS 16.0. All data are presented as means ± standard deviation (SD), with *p* < 0.05 indicating statistical significance. Because the levels of variance in serum TRACP 5b and L3 BV/TV at 4- and 8-week postoperation, L3 TbSp at 8- and 12-week postoperation, and femoral distal end TbSp at 12-week postoperaton were heterogeneous using Levene test, the Kruskal-Wallis H and Mann-Whitney U tests were used to analyze these data; other indicators, with homogenous variance, were analyzed using one-way analysis of variance (one-way ANOVA). 

## Results

### 1: BMD

**Figure 1:**
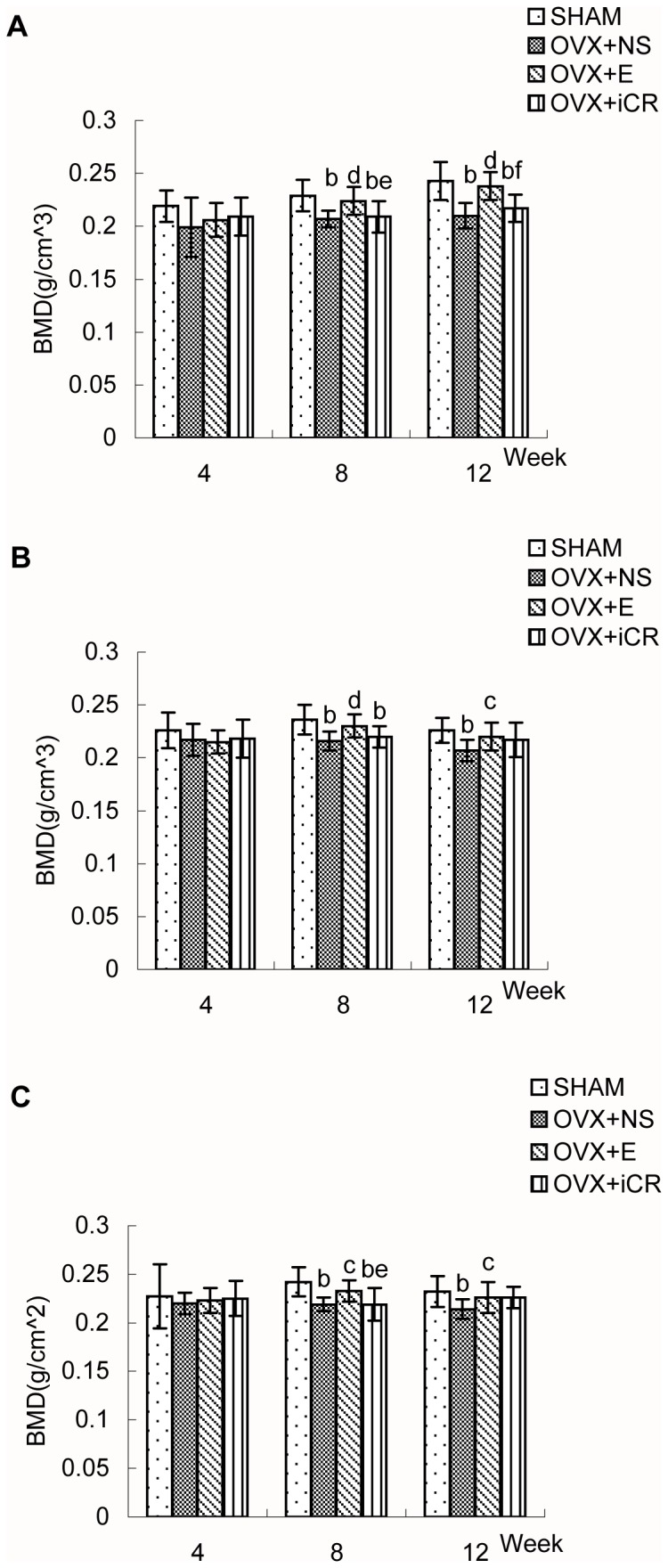
The BMD of lumbar vertebrae 2–4 (L2-L4) of the four groups. Comparison of bone mineral density (BMD) of lumbar vertebrae 2–4 (A:L2, B:L3, C:L4) in SHAM, OVX+NS, OVX+E and OVX+iCR groups at 4-, 8- and 12-week postoperation. Data represents the means ± standard deviation (SD ), n = 10 rats in each group. The x-axis represents time after surgery (weeks). Compared with SHAM group, a means *p*<0.05, b means *p*<0.01; Compared with OVX+NS group, c means *p*<0.05, d means *p*<0.01; Comparison between OVX+iCR and OVX+E group, e means *p*<0.05, f means *p*<0.01.

**Figure 2:**
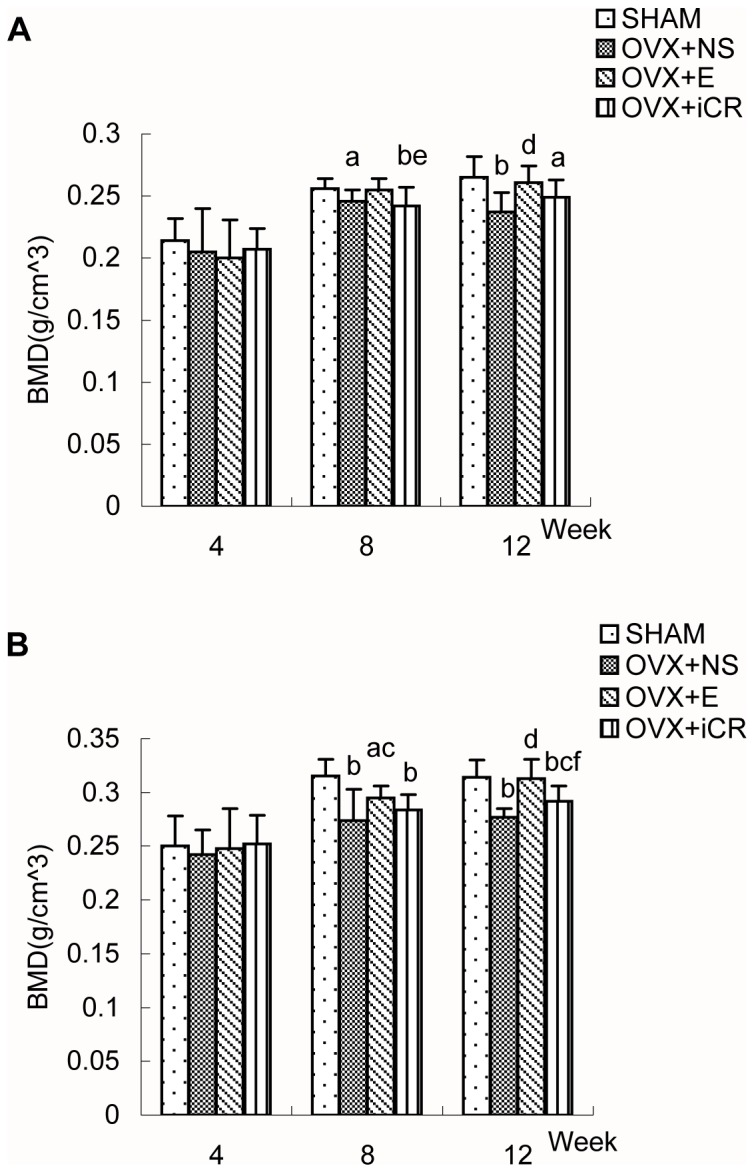
The BMD of femoral proximal and distal ends of the four groups. Comparison of bone mineral density (BMD) of femoral proximal ends (A) and distal ends (B) in SHAM, OVX+NS, OVX+E and OVX+iCR groups at 4-, 8- and 12-week postoperation. Data represents the means ± standard deviation (SD ), n = 10 rats in each group. The x-axis represents time after surgery (weeks). Compared with SHAM group, a means *p*<0.05, b means *p*<0.01; Compared with OVX+NS group, c means *p*<0.05, d means *p*<0.01; Comparison between OVX+iCR and OVX+E group, e means *p*<0.05, f means *p*<0.01.

Four weeks after surgery(Figure 1,2 and Table 1,2), there were no significant differences between the four groups in terms of BMD at the L2, L3, L4, or the femoral proximal or distal ends. 

**Table 1:** Comparison of the L2-L4 bone mineral densities of the four groups.

**Group**	**4-week postoperation**			**8-week postoperation**			**12-week postoperation**		
	L2BMD	L3BMD	L4BMD	L2BMD	L3BMD	L4BMD	L2BMD	L3BMD	L4BMD
SHAM	0.219±0.015	0.226±0.017	0.227±0.033	0.229±0.015	0.236±0.014	0.242±0.015	0.243±0.018	0.226±0.012	0.232±0.016
OVX+NS	0.199±0.028	0.217±0.015	0.220±0.011	0.207±0.008^b^	0.216±0.009^b^	0.219±0.007^b^	0.210±0.012^b^	0.207±0.010^b^	0.214±0.010^b^
OVX+E	0.206±0.016	0.215±0.011	0.223±0.003	0.224±0.013^d^	0.230±0.011^d^	0.233±0.011^c^	0.238±0.013^d^	0.220±0.013^c^	0.226±0.016^c^
OVX+iCR	0.209±0.018	0.218±0.018	0.225±0.018	0.209±0.015^be^	0.220±0.010^b^	0.219±0.017^be^	0.217±0.013^bf^	0.217±0.016	0.226±0.011

Comparison of bone mineral density (BMD, g/cm^3^) of the second, third and fourth lumbar vertebrae (L2BMD, L3BMD, L4BMD) in SHAM, OVX+NS, OVX+E and OVX+iCR groups at 4-, 8- and 12-week postoperation. Data represents the means ± standard deviation (SD ), n = 10 rats in each group. Compared with SHAM group, a means *p*<0.05, b means *p*<0.01; Compared with OVX+NS group, c means *p*<0.05, d means *p*<0.01; Comparison between OVX+iCR and OVX+E group, e means *p*<0.05, f means *p*<0.01.

**Table 2:** Comparison of the femoral proximal and distal ends bone mineral densities of the four groups.

Group	4-week postsurgery		8-week postsurgery		12-week postsurgery	
	pBMD	dBMD	pBMD	dBMD	pBMD	dBMD
SHAM	0.214±0.018	0.250±0.028	0.256±0.008	0.315±0.016	0.265±0.017	0.314±0.016
OVX+NS	0.205±0.035	0.242±0.023	0.246±0.009^a^	0.274±0.029^b^	0.237±0.016^b^	0.277±0.008^b^
OVX+E	0.200±0.031	0.248±0.037	0.255±0.009	0.295±0.011^ac^	0.261±0.013^d^	0.313±0.018^d^
OVX+iCR	0.207±0.017	0.252±0.027	0.242±0.015^be^	0.284±0.014^b^	0.249±0.014^a^	0.292±0.014^bcf^

Comparison of bone mineral density (BMD, g/cm^3^) of the femoral proximal (pBMD) and distal ends (dBMD) in SHAM, OVX+NS, OVX+E and OVX+iCR groups at 4-, 8- and 12-week postoperation. Data represents the means ± standard deviation (SD ), n = 10 rats in each group. Compared with SHAM group, a means *p*<0.05, b means *p*<0.01; Compared with OVX+NS group, *c* means *p*<0.05, d means *p*<0.01; Comparison between OVX+iCR and OVX+E group, e means *p*<0.05, f means *p*<0.01.

Eight weeks after surgery(Figure 1,2 and Table 1,2), ovariectomy significantly decreased the BMD of L2, L3, L4, the femoral proximal and distal ends while the BMD of the above test regions were significantly higher upon estradiol valerate treatment than in the OVX+NS group (*p* < 0.05 or 0.01). However, there was no statistical difference between OVX+NS group and OVX+iCR group in terms of BMD at the L2,L3, L4, or the femoral proximal or distal ends. The BMD of the test regions were ranked as follows: L2 BMD: SHAM = OVX+E > OVX+iCR = OVX+NS; L3 BMD: SHAM > OVX+E = OVX+iCR = OVX+NS, OVX+E > OVX+NS; L4 BMD: SHAM = OVX+E > OVX+iCR = OVX+NS; Femoral proximal end BMD: SHAM = OVX+E = OVX+NS = OVX+iCR, SHAM > OVX +NS, OVX+E > OVX+iCR; Femoral distal end BMD: SHAM > OVX+E = OVX+iCR = OVX+NS, OVX+E > OVX+NS.

Twelve weeks after surgery(Figure 1,2 and Table 1,2), the BMD of L2, L3, L4, femoral proximal and distal ends were reduced significantly by ovariectomy (*p* < 0.01) and were significantly higher upon estradiol valerate treatment (*p* < 0.05 or 0.01). Remifemin treatment, without significant effects on the BMD of L2, L3, L4 and femoral proximal end, resulted in a superior femoral distal end BMD (*p* < 0.05) when compared with OVX+NS group. The BMD of the test regions were ranked as follows: L2 BMD: SHAM = OVX+E > OVX+iCR = OVX+NS; L3 and L4 BMD: SHAM > OVX+E = OVX+iCR = OVX+NS, OVX+E > OVX+NS; Femoral proximal end BMD: SHAM > OVX+E = OVX+iCR = OVX+NS, SHAM > OVX+iCR, OVX+E > OVX+NS; Femoral distal end BMD: SHAM = OVX+E > OVX+iCR > OVX+NS.

All of the above differences are statistically significant, and the use of the equal sign should be regarded as statistically equivalent not mathematically equal (i.e., if A = B = C, then A = C or A > C or A < C). If not specifically mentioned, then A = C. 

### 2: Trabecular bone structure

#### 2.1: Trabecular bone structure of L3

**Figure 3:**
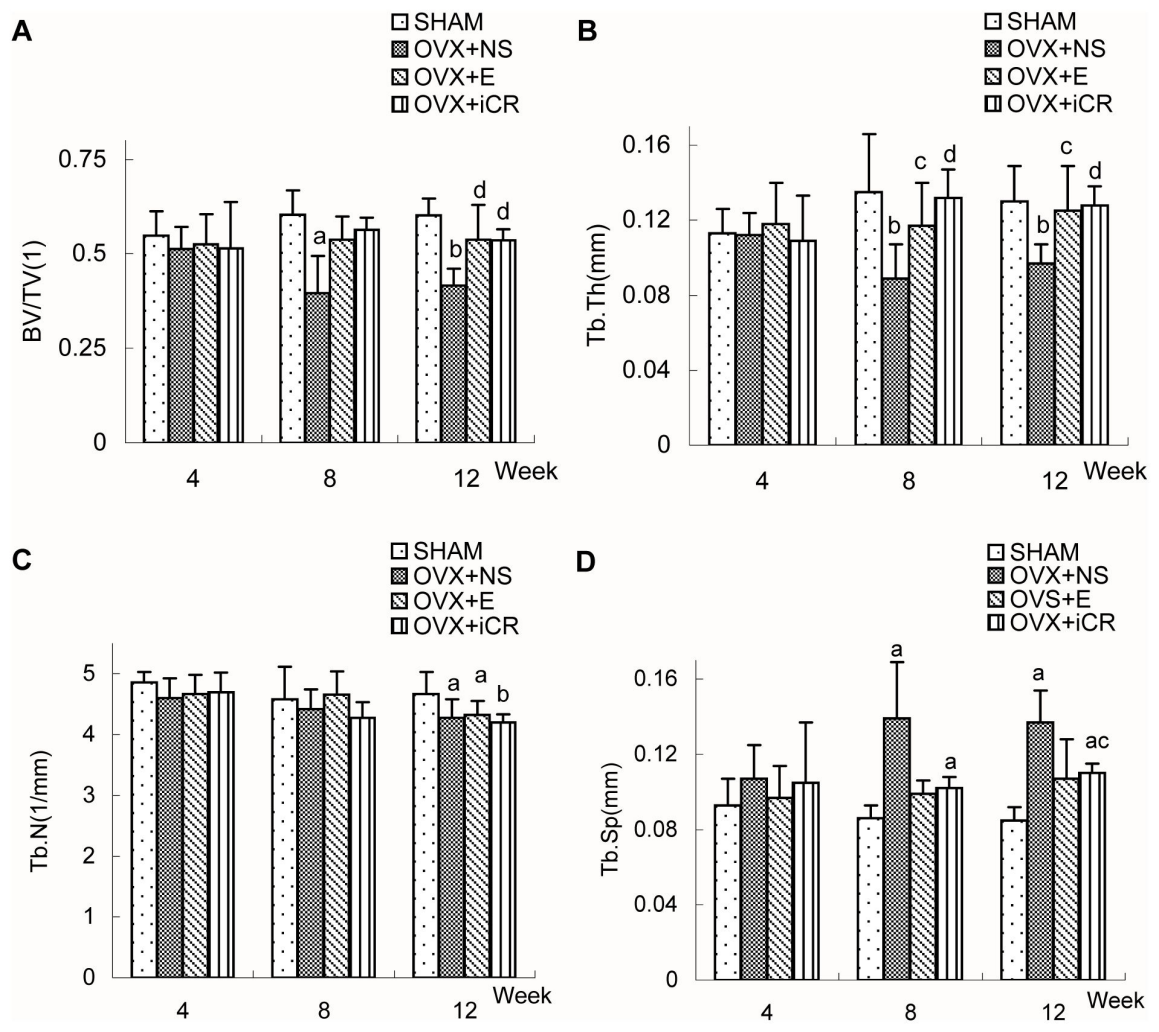
Comparisons of the morphometric indicators of L3 among the four groups. Comparison of bone morphometric indicators (relative bone volume (BV/TV), trabecular number (TbN), trabecular spacing (TbSp), and trabecular thickness (TbTh)) of the third lumbar vertebrae (L3) (A-D) in SHAM, OVX+NS, OVX+E and OVX+iCR groups at 4-, 8- and 12-week postoperation. Data represents the means ± standard deviation (SD), n=6 rats in each group. The x-axis represents time after surgery (weeks). Compared with SHAM group, a means *p*<0.05, b means *p*<0.01; Compared with OVX+NS group, c means *p*<0.05, d means *p*<0.01; Comparison between OVX+iCR and OVX+E group, e means *p*<0.05, f means *p*<0.01.

 Four weeks after surgery(Figure 3 and Table 3), there were no significant differences between the four groups in terms of the morphometric chacteristics of L3. 

**Table 3:** Comparisons of the morphometric indicators of the L3 and femoral distal end among the four groups at 4 weeks postoperation.

Group	BV/TV(1)		Tb.Th(mm)		Tb.N(1/mm)		Tb.Sp(mm)	
	L3	Femur	L3	Femur	L3	Femur	L3	Femur
SHAM	0.548±0.064	0.162±0.042	0.113±0.013	0.057±0.002	4.865±0.167	2.816±0.637	0.093±0.014	0.311±0.068
OVX+NS	0.513±0.058	0.169±0.043	0.112±0.012	0.057±0.005	4.602±0.329	2.965±0.542	0.107±0.018	0.290±0.061
OVX+E	0.525±0.080	0.162±0.036	0.118±0.022	0.061±0.004	4.674±0.313	2.634±0.454	0.097±0.017	0.328±0.066
OVX+iCR	0.514±0.124	0.160±0.033	0.109±0.024	0.057±0.003	4.697±0.325	2.786±0.425	0.105±0.032	0.308±0.052

Comparison of bone morphometric indicators (relative bone volume (BV/TV), trabecular number (TbN), trabecular spacing (TbSp), and trabecular thickness (TbTh)) of the L3 and femoral distal ends (Femur) in SHAM, OVX+NS, OVX+E and OVX+iCR groups at 4-week postoperation. Data represents the means ± standard deviation (SD), n=6 rats in each group. Compared with SHAM group, a means *p*<0.05, b means *p*<0.01; Compared with OVX+NS group, c means *p*<0.05, d means *p*<0.01; Comparison between OVX+iCR and OVX+E group, e means *p*<0.05, f means *p*<0.01.

Eight weeks after surgery(Figure 3 and Table 4), the OVX+NS group rats, without changes in TbN value, had lower value in BV/TV and TbTh and higher value in TbSp, when compared with SHAM group rats. Compared to the OVX+NS group, both estradiol valerate and Remifemin treatment resulted in a superior TbTh value.The four parameters of each group were ranked as follows: BV/TV: SHAM = OVX+iCR = OVX+E = OVX+NS, SHAM > OVX+NS; TbTh: SHAM = OVX+iCR = OVX+E > OVX+NS; there were no signifiant differences between the four groups in terms of TbN; TbSp was ranked as follows: OVX+NS =OVX+iCR = OVX+E =SHAM, OVX+NS =OVX+iCR >SHAM.

**Table 4:** Comparisons of the morphometric indicators of the L3 and femoral distal end among the four groups at 8 weeks postoperation.

Group	BV/TV(1)		Tb.Th(mm)		Tb.N(1/mm)		Tb.Sp(mm)	
	L3	Femur	L3	Femur	L3	Femur	L3	Femur
SHAM	0.604±0.064	0.196±0.018	0.135±0.031	0.062±0.008	4.584±0.532	3.223±0.401	0.086±0.007	0.253±0.037
OVX+NS	0.396±0.099^a^	0.142±0.015^b^	0.089±0.018^b^	0.058±0.003	4.417±0.332	2.475±0.305^b^	0.139±0.030^a^	0.346±0.045^b^
OVX+E	0.538±0.061	0.191±0.025^d^	0.117±0.023^c^	0.062±0.004	4.662±0.378	3.074±0.247^d^	0.099±0.007	0.265±0.030^d^
OVX+iCR	0.563±0.033	0.180±0.024^d^	0.132±0.015^d^	0.067±0.004^d^	4.276±0.264	2.678±0.300^be^	0.102±0.006^a^	0.310±0.037^a^

Comparison of bone morphometric indicators (relative bone volume (BV/TV), trabecular number (TbN), trabecular spacing (TbSp), and trabecular thickness (TbTh)) of the L3 and femoral distal ends (Femur) in SHAM, OVX+NS, OVX+E and OVX+iCR groups at 8-week postoperation. Data represents the means ± standard deviation (SD), n=6 rats in each group. Compared with SHAM group, a means *p*<0.05, b means *p*<0.01; Compared with OVX+NS group, c means *p*<0.05, d means *p*<0.01; Comparison between OVX+iCR and OVX+E group, e means *p*<0.05, f means *p*<0.01.

Twelve weeks after surgery(Figure 3 and Table 5), compared to the SHAM group rats, the OVX+NS group rats had lower value in BV/TV, TbTh, TbN and higher value in TbSp. Both estradiol valerate and Remifemin treatment resulted in a superior BV/TV and TbTh value when compared with OVX+NS group. what’s more, Remifemin treatment decreased the TbSp value. The four parameters of each group were ranked as follows: BV/TV: SHAM = OVX+iCR = OVX+E > OVX+NS; TbTh: SHAM = OVX+iCR = OVX+E > OVX+NS; TbN: SHAM > OVX+E = OVX+NS = OVX+iCR; TbSp: OVX+NS > OVX+iCR > SHAM, the OVX+E group demonstrated no statistically significant differences in comparison with the other three groups. 

**Table 5:** Comparisons of the morphometric indicators of the L3 and femoral distal end among the four groups at 12 weeks postoperation.

Group	BV/TV(1)		Tb.Th(mm)		Tb.N(1/mm)		Tb.Sp(mm)	
	L3	Femur	L3	Femur	L3	Femur	L3	Femur
SHAM	0.602±0.045	0.198±0.033	0.130±0.019	0.061±0.005	4.670±0.361	3.265±0.329	0.085±0.007	0.247±0.033
OVX+NS	0.416±0.044^b^	0.117±0.021^b^	0.097±0.010^b^	0.060±0.004	4.282±0.298^a^	1.956±0.342^b^	0.137±0.017^a^	0.464±0.089^a^
OVX+E	0.537±0.092^d^	0.194±0.035^d^	0.125±0.024^c^	0.068±0.007	4.328±0.230^a^	2.864±0.379^d^	0.107±0.021	0.287±0.047^c^
OVX+iCR	0.536±0.029^d^	0.188±0.030^d^	0.128±0.010^d^	0.071±0.009^ac^	4.203±0.132^b^	2.663±0.302^bd^	0.110±0.005^ac^	0.309±0.042^c^

Comparison of bone morphometric indicators (relative bone volume (BV/TV), trabecular number (TbN), trabecular spacing (TbSp), and trabecular thickness (TbTh)) of the L3 and femoral distal ends (Femur) in SHAM, OVX+NS, OVX+E and OVX+iCR groups at 12-week postoperation. Data represents the means ± standard deviation (SD), n=6 rats in each group. Compared with SHAM group, a means *p*<0.05，b means *p*<0.01; Compared with OVX+NS group, c means *p*<0.05, d means *p*<0.01; Comparison between OVX+iCR and OVX+E group, e means *p*<0.05, f means *p*<0.01.

#### 2.2: Trabecular bone structure of the femoral distal end

**Figure 4:**
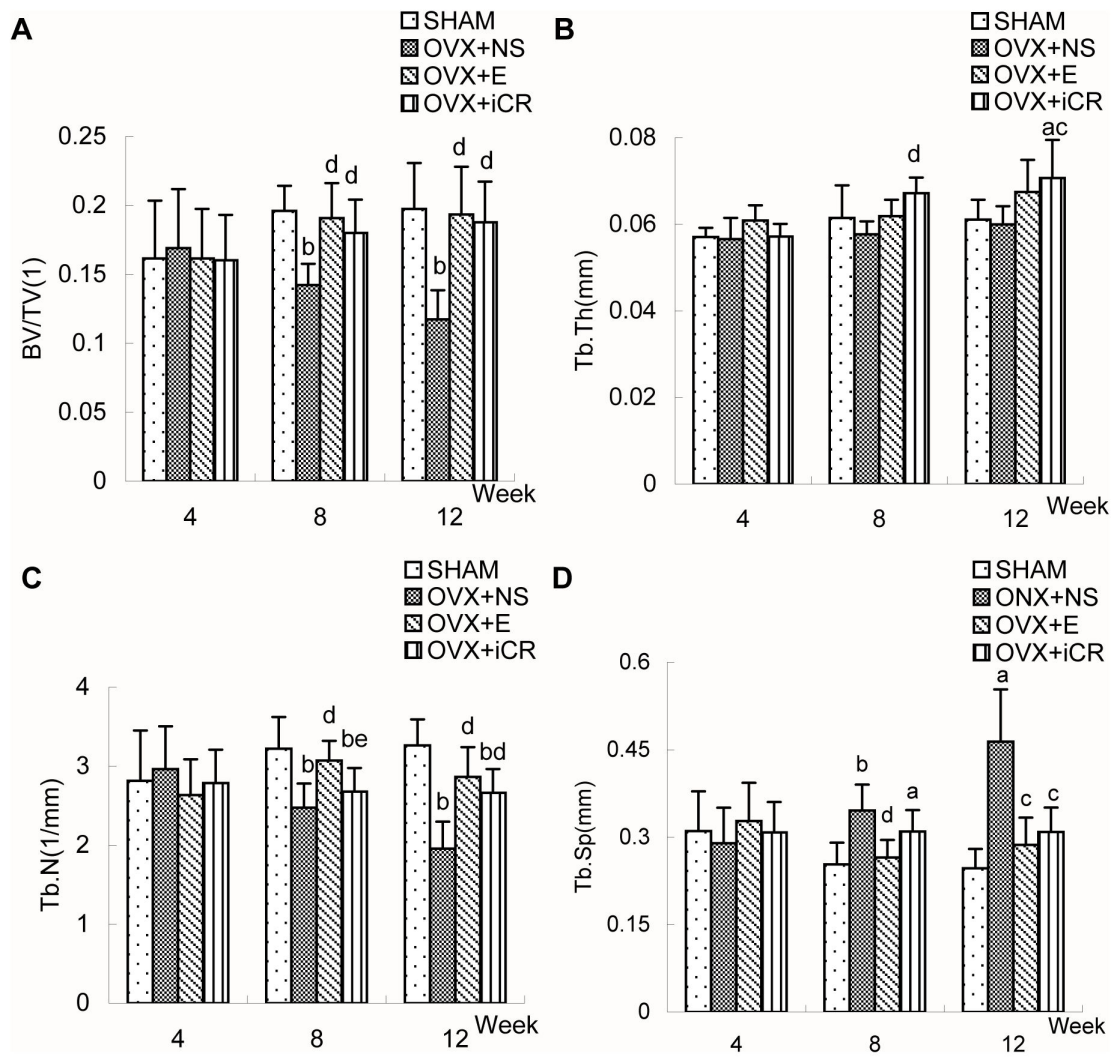
Comparisons of the morphometric indicators of the femoral distal end among the four groups. Comparison of bone morphometric indicators (relative bone volume (BV/TV), trabecular number (TbN), trabecular spacing (TbSp), and trabecular thickness (TbTh)) of the femoral distal ends (A-D) in SHAM, OVX+NS, OVX+E and OVX+iCR groups at 4-, 8- and 12-week postoperation. Data represents the means ± standard deviation (SD), n=6 rats in each group. The x-axis represents time after surgery (weeks). Compared with SHAM group, a means *p*<0.05, b means *p*<0.01; Compared with OVX+NS group, c means *p*<0.05, d means *p*<0.01; Comparison between OVX+iCR and OVX+E group, e means *p*<0.05, f means *p*<0.01.

Four weeks after surgery(Figure 4 and Table 3), there were no significant differences between the four groups in terms of the morphometric indicators.

Eight weeks after surgery(Figure 4 and Table 4), the OVX+NS group rats, without changes in TbTh value, had lower value in BV/TV, TbN and higher value in TbSp, when compared with SHAM group rats. Estradiol valerate treatment resulted in a higher BV/TV, TbN value and lower TbSp value while Remifemin treatment resulted in a higher BV/TV and TbTh value, without changes in the TbN and TbSp value, when compared with OVX+NS group. The four parameters of each group were ranked as follows:BV/TV: SHAM = OVX+E = OVX+iCR > OVX+NS; TbTh: OVX+iCR = OVX+E = SHAM = OVX+NS, OVX+iCR > OVX+NS; TbN: SHAM = OVX+E > OVX+iCR = OVX+NS; TbSp: SHAM = OVX+E = OVX+iCR = OVX+NS, OVX+iCR > SHAM, OVX+NS > OVX+E = SHAM. 

Twelve weeks after surgery(Figure 4 and Table 5), ovariectomy decreased the BV/TV, TbN value and increased the TbSp value, demonstrating a loss of trabeculae in femoral distal end. Both estradiol valerate and Remifemin treatment resulted in a higher BV/TV and TbN value and lower TbSp value, while Remifemin treatment also resulted in a superior TbTh value, when compared with OVX+NS group. The four parameters of each group were ranked as follows: BV/TV: SHAM = OVX+E = OVX+iCR > OVX+NS; TbTh: OVX+iCR = OVX+E = SHAM = OVX+NS, OVX+iCR > SHAM, OVX+iCR > OVX+NS; TbN: SHAM = OVX+E = OVX+iCR > OVX+NS, SHAM > OVX+iCR; TbSp: OVX+NS > OVX+iCR = OVX+E = SHAM.

**Figure 5:**
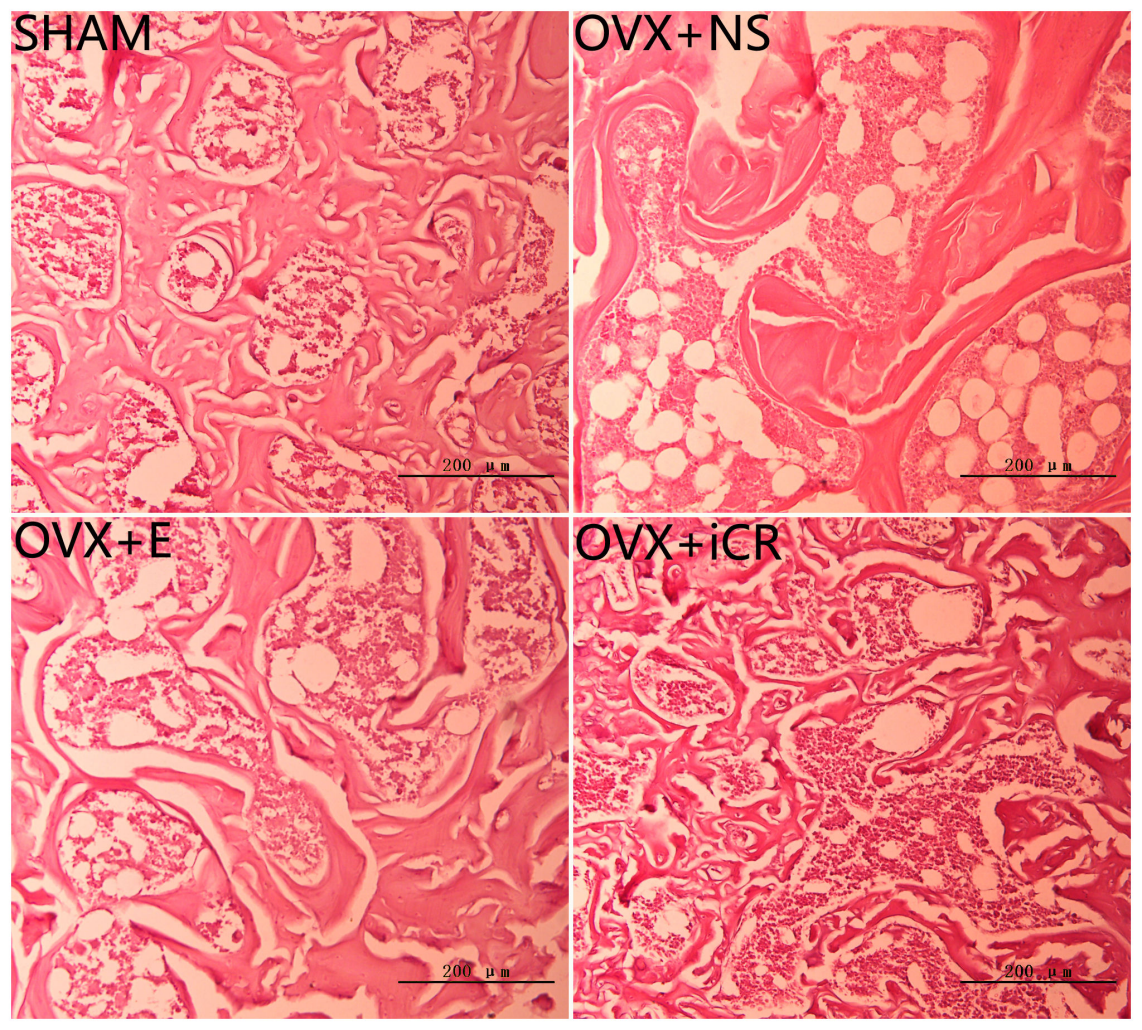
H&E staining of trabecular bone from the left femoral distal ends at 12-week postoperation. Histological sections (stained with H&E) of trabecular bone from the left femoral distal ends of the four groups at 12 weeks after surgery. The OVX+NS group rats showed sparse and thinning trabeculae, with loss of connectivity and presence of abundant adiposity cells, while estradiol valerate and Remifemin treatment significantly protected trabecular bone from ovariectomy-induced osteopenia.

The above changes of trabeculae in femoral distal end at 12-week postoperation could also be seen in the left femoral distal end H&E staining (Figure 5 ). The OVX+NS group rats showed sparse and thinning trabeculae, with loss of connectivity and presence of abundant adiposity cells, while estradiol valerate and Remifemin treatment significantly protected trabecular bone from ovariectomy-induced osteopenia. 

All of the above differences are statistically significant, and the use of the equal sign should be regarded as statistically equivalent not mathematically equal (i.e., if A = B = C, then A = C or A > C or A < C). If not specifically mentioned, then A = C. 

### 3: Bone biomechanical properties

**Figure 6:**
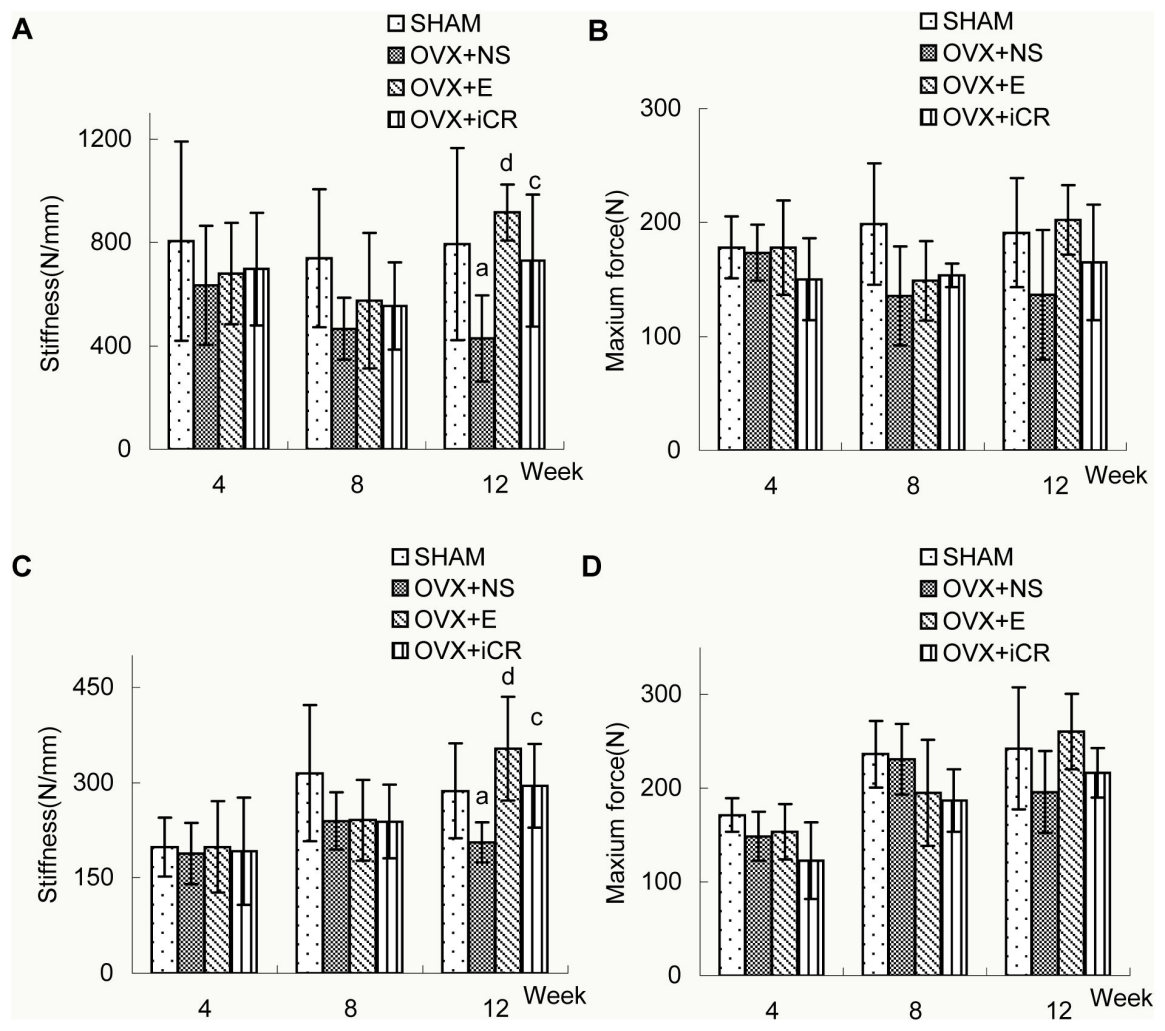
Changes in biomechanical parameters at L3 and the femoral distal end among the four groups. Biomechanical changes in bone stiffness and bone strength at L3 (A-B) and the femoral distal end (C-D) in SHAM, OVX+NS, OVX+E and OVX+iCR groups at 4-, 8- and 12-week postoperation. Data represents the means ± standard deviation (SD), n = 6 rats in each group. The x-axis represents time after surgery (weeks). Compared with SHAM group, a means *p*<0.05, b means *p*<0.01; Compared with OVX+NS group, c means *p*<0.05, d means *p*<0.01.

There were no significant differences between the four groups in terms of bone stiffness in the L3 and femoral distal end at 4- or 8-week postoperation. Twelve weeks after surgery, bone stiffness of the L3 and femoral distal end in the OVX+E, OVX+iCR, and SHAM groups were statistically the same; however, bone stiffness in the OVX+NS group was significantly lower than the other three groups. In terms of bone strength, there were no significant differences between the four groups at 4- , 8- or 12-week postoperation, however, a modest superiority of bone strength in L3 and femoral distal end at 12-week postoperation in comparison with OVX+NS group was obtained, though without statistical significance. See Figure 6 and Table 6,7.

**Table 6:** Comparisons of biomechanical parameters at L3 among the four groups at 4-, 8- and 12-week postoperation.

Group	4-week postoperation		8-week postoperation		12-week postoperation	
	Stiffness	Maximun force	Stiffness	Maximun force	Stiffness	Maximun force
SHAM	806.06±385.33	178.17±27.10	739.04±267.86	198.50±53.22	793.53±372.56	191.03±48.01
OVX+NS	633.96±230.66	173.50±24.70	466.29±120.20	135.33±43.43	428.05±166.72	136.58±57.05^a^
OVX+E	679.56±195.79	178.00±41.52	575.23±262.26	148.83±34.79	915.85±107.66	202.07±30.54^d^
OVX+iCR	696.97±218.53	150.17±35.83	554.96±169.06	153.67±10.23	729.65±256.09	164.78±50.72^c^

Biomechanical changes in bone stiffness and bone strength at L3 in SHAM, OVX+NS, OVX+E and OVX+iCR groups at 4-, 8- and 12-week postoperation. Data represented the means ± standard deviation (SD), n = 6 rats in each group. Compared with SHAM group, a means *p*<0.05, b means *p*<0.01; Compared with OVX+NS group, c means *p*<0.05, d means *p*<0.01.

**Table 7:** Comparisons of biomechanical parameters at the femoral distal end among the four groups 4-, 8- and 12-week postoperation.

Group	4-week postoperation		8-week postoperation		12-week postoperation	
	Stiffness	Maximun force	Stiffness	Maximun force	Stiffness	Maximun force
SHAM	198.37±46.54	171.52±18.22	314.84±107.17	236.66±35.57	287.09±75.11	242.39±65.18
OVX+NS	187.91±48.30	148.64±26.14	239.51±44.92	231.03±37.55	205.93±31.75	195.90±43.63^a^
OVX+E	198.74±72.07	153.67±29.70	240.94±63.71	195.04±56.59	353.67±81.52	260.66±40.49^d^
OVX+iCR	192.04±84.75	122.80±41.03	238.49±57.97	186.98±33.62	294.89±66.13	216.49±26.44^c^

Biomechanical changes in bone stiffness and bone strength at the femoral distal end in SHAM, OVX+NS, OVX+E and OVX+iCR groups at 4-, 8- and 12-week postoperation. Data represented the means ± standard deviation (SD), n = 6 rats in each group. Compared with SHAM group, a means *p*<0.05, b means *p*<0.01; Compared with OVX+NS group, c means *p*<0.05, d means *p*<0.01.

### 4: Bone turnover markers in serum

**Figure 7:**
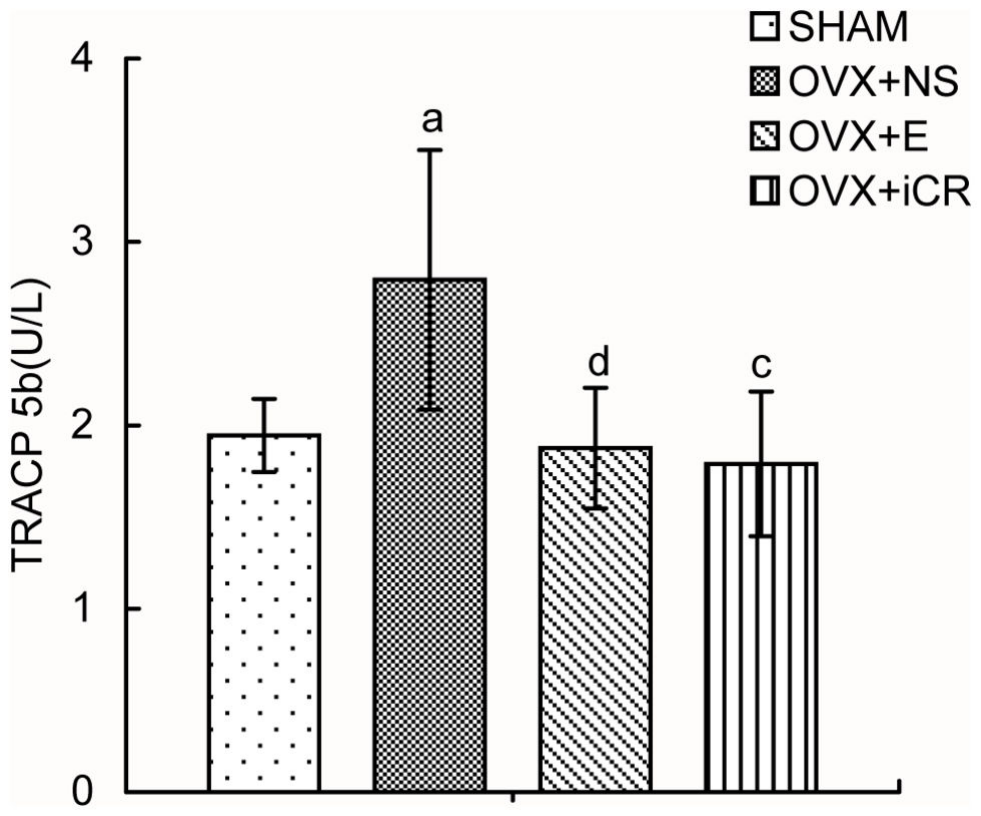
Comparison of TRACP 5b levels among the four groups at 12-weeks postoperation. The serum tartrate-resistant acid phosphatase 5b (TRACP 5b) level of SHAM, OVX+NS, OVX+E and OVX+iCR groups at 12-week postoperation were determined using ELISA kit. Data represents the means ± standard deviation (SD), n=10 rats in each group. Compared with SHAM group, a means *p*<0.05, b means *p*<0.01; Compared with OVX+NS group, c means *p*<0.05, d means *p*<0.01.

There was a significant difference in terms of the serum TRACP 5b level between OVX+NS and SHAM group, while treatment with estradiol valerate or Remifemin restored TRACP 5b levels up to those of the SHAM group (Figure 7 and Table 8). There were no statistical differences between the four groups in terms of OC or PINP (data not shown).

**Table 8:** Comparison of TRACP 5b levels at 12-week postoperation.

Group	SHAM	OVX+NS	OVX+E	OVX+iCR
TRACP 5b (U/L)	1.945 ± 0.199	2.793 ± 0.708^a^	1.876 ± 0.329^d^	1.790 ± 0.395^c^

The serum tartrate-resistant acid phosphatase 5b (TRACP 5b) level of SHAM, OVX+NS, OVX+E and OVX+iCR groups at 12-week postoperation was determined using ELISA kit. Data represented the means ± standard deviation (SD), n=10 rats in each group.Compared with SHAM group, a means *p*<0.05，b means *p*<0.01; Compared with OVX+NS group, c means *p*<0.05，d means *p*<0.01.

## Discussion

The use of HRT has encountered great challenges since the publication of the Women's Health Initiative (WHI) research results in 2002, and the use of non-HRT for the treatment of the symptoms of menopause has recently become an important research topic. Clinically, the therapeutic effects of Remifemin on menopausal symptoms, such as hot flushes, sweatings and other symptoms, have been confirmed [21,23]. However, there is still no method for evaluating the systematic effects of Remifemin on postmenopausal osteoporosis. Therefore, the aims of the current study were to investigate the effects of Remifemin on the prevention of postmenopausal osteoporosis in an ovariectomized rat model [32] upon intragastric administration. The evaluation comprised BMD, trabecular bone structure, the biomechanical properties of weight-bearing and non-weight-bearing bone, and bone formation and resorption. 

Osteoporosis is a metabolic disorder in which the loss of bone mass and strength leads to fragility fractures [33]. In addition, the deterioration of the trabecular architecture has been implicated in decreased bone strength and the incidence of fracture [1]. Therefore, BMD, trabecular structure, and bone mechanics were chosen as indicators of osteoporosis in the current study. Femoral diaphysis mainly develops in cortical bone, while the femoral distal and proximal ends consist of trabecular bone (particularly the femoral distal end where trabecular bone is prominent [31]. The lumbar vertebra is also rich in trabecular bone. Because trabecular bone is more readily lost in our animal model following ovariectomy [34-36], the femoral distal and proximal ends and L2-L4 were chosen for BMD testing. 

Eight weeks after ovariectomy, the BMD values of these regions decreased considerably. The adminstration of estradiol valerate significantly prevented this deterioration, indicating that estrogen is important for maintaining bone density and that low estrogen levels accelerate bone loss after menopause [6]. Remifemin has demonstrated the ability to significantly inhibit the decrease in BMD in the femoral distal end following ovariectomy; however, the onetime effectiveness of Remifemin is weaker and occurs later compared with estradiol valerate. The BMD values of the femoral proximal end and L3-L4 in OVX+iCR group, demonstrating no significant differences compared with the OVX+NS group, were approaching the levels of the SHAM and OVX+E groups gradually with an extended course of Remifemin treatment, implying a slow onset of Remifemin effects on BMD. 

The effectiveness of Remifemin was more prominent in weight-bearing bones, and equivalent bone-biomechanical effectiveness was achieved in both weight-bearing and non-weight-bearing bones upon extended treatment. Nißlein et al reported that following 12 weeks of treatment with Remifemin, the difference in L3 BMD values between the Remifemin and ovariectomy groups was small according to the pQCT method, but significant according to the Archimedes method [27]. In the current study, dual-energy X-ray absorptiometry was used for BMD testing, and the results may have varied due to different methods.

According to the BMD results, the femoral distal end (weight-bearing bone) and L3 (non-weight-bearing bone), which were sensitive to Remifemin treatment, were analyzed using Micro CT scanning and compression testing. The H&E staining of the left femoral distal end was also applied to further evaluate the trabecular bone structure. MicroCT was recently developed as a method that can quickly evaluate the morphology and structure of trabecular bone [37]. In the current study, evaluation of the trabecular bone at the L3 and femoral distal end using MicroCT indicated that ovariectomy significantly reduced trabecular BV/TV and TbTh (or TbN) but increased TbSp, which are typical changes seen in cancellous bone loss. The H&E staining of the left femoral distal end conveyed the same information as Micro CT. Both estradiol valerate and Remifemin significantly ameliorated alteration of the trabecular structure, though the time of onset of the effects and their profile may not be completely uniform between Remifemin and estrogen; in addition, the effects of Remifemin on weight-bearing bone and non-weight-bearing bone were not exactly the same. The MicroCT and BMD results both indicate that Remifemin demonstrates a stronger effect on the trabecular architecture of weight-bearing bone than non-weight-bearing bone. Although both estradiol valerate and Remifemin can, to some degree, inhibit damage to trabecular bone, they cannot restore deterioration, which is consistent with the results of other published studies [38,39], indicating that preventing the deterioration of trabecular bone is far more important than treatment. 

Biomechanical analysis of bone can provide important information regarding the integrity of bone function [40]. Bone stiffness and strength were assessed in this study in order to evaluate the biomechanical properties of L3 and the femoral distal end. Bone stiffness was defined by the extent to which bone deforms under a particular load [41] and bone strength was defined by the ultimate load that factures bone tissue, which is closely related to BMD, bone structure, bone connection, bone mineralization, etc [41,42]. Both indicators reflect the bone’s properties at the organ level [37]. Our findings indicate that the bone stiffness of the lumbar vertebrae and femoral distal end decreased along with BMD, resulting in the deterioration of the trabecular bone structure in ovariectomized rats. Estradiol valerate significantly inhibited this decrease in BMD and subsequent damage to the trabecular bone structure, thereby significantly increasing the bone stiffness of the lumbar vertebrae and femoral distal end. Although the effects of Remifemin were slightly weaker than estradiol valerate in terms of BMD, its overt beneficial effects on the trabecular bone of the lumbar vertebrae and femoral distal end make its effects on bone stiffness even more important. Although low bone-mass is the main risk factor for fracture, the preservation of the trabecular microstrucure may improve the biomechanical properties of bone better than just increasing BMD or bone mineral content[35]. In the current study, rats that received Remifemin or estradiol valerate demonstrated a modest improvement in bone strength compared with rats that did not receive medication, which was unexpected. And the modesty might be related to the short observational period. Hence, the effects of these drugs may not have been fully exerted, yet.

The decrease in BMD and the deterioration of bone microarchitecture induced by the cessation of ovarian function are closely associated with a high bone remodeling rate, which leads to imbalance between bone formation and resorption (i.e., osteoclast cell-mediated bone resorption is higher than osteoblast-mediated bone formation, resulting in low bone-mass, high fragility, and the high incidence of fracture) [1,7]. Bone turnover markers are widely used to measure the effects of various drugs on bone remodeling [43]. Serum TRACP 5b, a subtype of TRACP 5, is secreted only by osteoclasts; therefore, its activity can be used as an indicator of osteoclast activity and bone reabsorption [44]. Serum TRACP 5b increased significantly along with bone reabsorption in ovariectomized rats, which is consistent with our measurements of BMD and trabecular bone. Treatment with estradiol valerate or Remifemin significantly inhibited TRACP 5b, indicating the inhibition of bone resorption. On the other hand, although serum OC and PINP are regarded as sensitive indicators of bone formation, they were not significantly altered in the four groups included in the current study. This may be due to loss of the bone formation template, which is massively damaged as bone reabsorption increases following ovariectomy [45]. When drug therapy was applied, the bone turnover rate was inhibited and the bone formation rate decreased accordingly, so no statistical variations in the bone formation markers were found between the four groups. In addition, an *in vitro* study reported that Remifemin promoted the production of osteoprotegerin in osteoblasts and increased the serum concentration of OC and bone alkaline phosphatase, both of which could promote bone formation [46]. However, further studies on the association between Remifemin and bone formation are needed.

Because certain extracts of *Cimicifuga Racemosa* are already recognized as safe and useful for the treatment of certain gynecological disorders [20,27], this study did not include an assessment of the safety of Remifemin; besides, due to the short duration of this experiment and the small number of animals, we were unable to observe the maximum effects of this drug. More studies are needed to fully elucidate Remifemin’s specific mechanisms that affect bone protection, and also regarding its optimal treatment duration and dosage for this additional benefit. 

## Conclusions

Remifemin demonstrated similar effects as estradiol valerate in preventing damage to trabecular bone, improving the biomechanical properties of bone, and inhibiting bone reabsorption in ovariectomized rats. By extending the duration of treatment, the preventative effects of Remifemin on bone loss can be similar to those of estradiol valerate. In conclusion, Remifemin, with a better safety profile, may demonstrate equivalent effects as estradiol valerate in terms of preventing postmenopausal osteoporosis. This study provides strong evidence that Remifemin could be used for the prevention of clinical PMO. 
